# Attentional Focus Instructions Do Not Affect Choice Reaction Time

**DOI:** 10.3389/fpsyg.2021.675641

**Published:** 2021-05-10

**Authors:** Gal Ziv, Ronnie Lidor

**Affiliations:** Motor Behavior Laboratory, The Academic College at Wingate, Netanya, Israel

**Keywords:** attentional focus, choice reaction time, online studies, inhibition, Simon task

## Abstract

The majority of the studies on attentional focus have shown that participants who were instructed to focus externally performed better than those who were taught to focus internally. However, in most of these studies the participants performed complex motor tasks. Due to the scarcity of data on the effects of attentional focus specifically on simple motor tasks, our purpose in the current study was to examine these effects on two simple reaction time (RT) tasks. The study was conducted on a cloud-based experimental software. Participants were allocated to three experimental groups: an external focus group (*n* = 44), an internal focus group (*n* = 46), and a control group (no attentional instructions; *n* = 47). The participants performed two tasks: a choice-RT task and a Simon task. Participants in all three groups practiced eight blocks of 20 trials from each task in a counterbalanced order – a total of 180 trials for each task. The sole difference between the three groups was the administered attentional focus instructions. The findings suggest that attentional focus instructions do not affect the performance of a choice-RT task or a Simon-task in a computerized online study. It is possible that the simple RT-based tasks in the current study were not sensitive to the attentional focus manipulation, since in such simple tasks there are not many actions that internal focus can disrupt. Although we asked the participants to what extent they followed the instructions, we cannot say whether their responses represent their actual attentional focus when performing the tasks.

## Introduction

The effects of external and internal focus of attention on motor performance and learning have been researched extensively over the past two decades. When performing a motor task, we can focus our attention internally – to our body movements, or externally – to the movement’s effect or to the object manipulated by the movement (Wulf, 2013). Most studies suggest that external focus of attention facilitates performance and learning, whereas internal focus of attention does not (for a review, see Wulf, 2013). These studies introduced a number of motor tasks to the participants, among them golf putting (e.g., Kearney, 2015), dart throwing (e.g., Lohse et al., 2010), balancing (e.g., Chiviacowsky et al., 2010), soccer kicking (e.g., Wulf et al., 2002, Exp. 2), and basketball free-throw shooting (e.g., Zachry et al., 2005). The benefits of following an external focus of attention have been previously explained by the constrained action hypothesis. According to this hypothesis, adopting an internal focus of attention disrupts automatic motor control processes, but this does not occur when adopting an external focus of attention (Wulf and Lewthwaite, 2016).

Despite the abundance of studies that support the beneficial effects of external focus of attention on performance and learning, some studies show that this may not always be the case. For example, Perkins-Ceccato et al. (2003) showed that external focus of attention benefited highly skilled golfers, but that internal focus of attention benefited low-skilled golfers. In another study (Wulf, 2008), acrobats performed better in a balancing task under a control condition compared to both external and internal instruction conditions. Finally, Rienhoff et al. (2015) found that an external focus of attention led to reduced basketball free-throw shooting performance and to sub-optimal gaze behavior in novice, advanced, and expert female basketball players.

Regardless of the effects of the different types of attentional foci on performance, all the above-mentioned tasks share a common denominator – they are all relatively complex tasks that either require accuracy and/or are performed by using several degrees of freedom. The question we ask, then, is: Do focus of attention instructions affect simple tasks, such as reaction time (RT) tasks, as well? The effects of different attentional foci (e.g., sensory vs. motor) on RT were examined several decades ago (e.g., Henry, 1960; Christina, 1973; Wrisberg and Pushkin, 1976). Henry (1960) and Christina (1973), for example, showed that attention toward the movement to be made (similar to internal focus of attention) led to longer RTs compared with attention toward the stimulus to respond to (e.g., sensory attention – different from the current definition of external focus that relates to the movement’s effect or to the object manipulated by the movement).

Although the abovementioned studies examined the effects of different attentional foci on RT, only a small number of studies examined the specific effects of external and internal focus of attention (as more recently defined) on the performance of simple tasks or on RT. For example, Porter and Anton (2011) showed that older adults undergoing chemotherapy performed a rotary pursuit task better when using external focus instructions compared with internal focus instructions or no instructions. Raisbeck et al. (2020) found benefits for external focus of attention in a reciprocal aiming task with two levels of difficulty. In addition, Ille et al. (2013) reported faster sprint start RTs under external focus conditions compared with internal focus conditions in both novices and experts. In contrast, Gottwald et al. (2019; Exp. 1) reported that, compared to external focus of attention, using internal focus of attention led to more accurate movements in a computer-based aiming movement task. Finally, Zimmermann et al. (2012) examined neural correlates of attentional focus in a Functional Magnetic Resonance Imaging scanner and found neural modulation when changing from external to internal focus or vice versa. However, there were no behavioral differences in a finger tapping task.

Our purpose in the current study was to examine the effects of external and internal focus of attention instructions on two simple RT-based tasks: (a) a choice-RT task, and (b) a Simon task. Based on the majority of studies on attentional focus, we hypothesized that external focus of attention instructions will lead to better performance in simple motor tasks compared to internal focus of attention instructions or to no instructions.

## Materials and Methods

### Pre-registration and Raw Data Repository

The study’s main question, experimental conditions, methodology, power analysis, dependent variables, and data analyses were all pre-registered on AsPredicted^1^ and can be accessed online.^2^ One change from the pre-registration is that the term *retention test* in the pre-registration was changed to *post-test*. Any other deviations from the pre-registered information are reported in the manuscript. The raw dataset can be accessed on OSF.^3^

### Experimental Approach

The study was conducted on a cloud-based experimental software (Anwyl-Irvine et al., 2020).^4^ This web-based software allows researchers to design online experiments, and enables individuals to participate in such experiments using their own computers. Our main measure was RT; it has been shown that web-based RT measurements are comparable to lab-based measurements (e.g., Crump et al., 2013; Schubert et al., 2013; Simcox and Fiez, 2014; Hilbig, 2016). For example, Hilbig (2016) reported similar mean RTs in a lexical decision task (a) on an experimental software in a laboratory setting, (b) on the same computer using a similar task written in HTML with RTs measured using JavaScript, and (c) when using the same HTML program, but on a web browser on any computer.

### Participants

We recruited participants through Prolific^5^ – an online platform that allows individuals with access to the Internet to participate in online studies. Each participant was paid 2.5 British Pounds for his or her participation in our 20-min study. We used G*Power software (Faul et al., 2007) to calculate a sample size, based on alpha = 0.05, effect size *f* = 0.25 (moderate effect), and 80% statistical power. Under these conditions, 120 participants are required to find group differences in a two-way ANOVA. For the repeated measures factor and for the interaction, the required sample sizes are much lower.

In such an online study, we are not able to know at the outset if participants who begin the study will decide to withdraw before completing it. Therefore, we recruited 140 participants to ensure that we would have the required sample size of 120 participants. Most of the participants completed the study, and we ended up with a sample size of 137 participants between the ages of 18 and 35 years. Using automatic, simple computerized randomization, the software allocated participants to three experimental groups: (a) an external focus group (*n* = 44), (b) an internal focus group (*n* = 46), and (c) a control group (no attentional instructions; *n* = 47). The inequality in the number of participants in each group is due to the automatic randomization process, which sometimes fails to account for participants who began their participation but withdrew before completing the study. All the participants filled out an electronic informed consent form prior to participation, and the study was approved by the ethics committee of the Academic College at Wingate (approval # 279).

### Tasks

The participants performed two tasks: A choice-RT task and a Simon task.

#### Choice-RT Task

**Figure 1:**
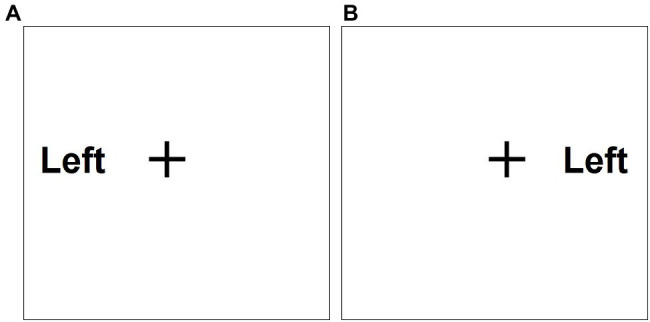
An example of the choice-reaction time (RT) task **(A)** and the Simon task **(B)**.

In this task, the participants pressed as quickly as possible the “j” key if the word “right” appeared on the right side and the “f” key if the word “left” appeared on the left side of a centralized cross on the computer screen. The words “right” or “left” were presented for 900 ms, followed by 600 ms during which only the centralized cross was displayed (see Figure 1A; Smith, 1968; Burle et al., 2004).

#### Simon Task

This task differed from the choice-RT task in one aspect: the words “right” or “left” could be displayed on either the right or the left side of the cross. The participants were required to press the “j” key if they saw the word “right” (even if it appeared on the left side of the cross) and to press the “f” key if they saw the word “left” (even if it appeared on the right side of the cross; see Figure 1B; Simon and Wolf, 1963; Lu and Proctor, 1995).

### Procedure

After reading the online consent form and agreeing to participate in the study, the participants were familiarized with the two tasks in a counterbalanced order. The participants performed eight trials of both the choice-RT task and the Simon task. Then, the online software randomized the participants to the three experimental groups. Participants in all three groups practiced eight blocks of 20 trials from each task – a total of 180 trials for each task. They completed all eight blocks of one task before continuing to the second task, but the order of tasks was counterbalanced. The only difference between the groups was the administered attentional focus instructions.

In each of the tasks, before practice began, the participants in the external focus group were given the following instructions: “Focus your attention on *pressing the relevant key* on your keyboard (“f” or “j”) as fast and as accurately as possible.” Participants in the internal focus group were given different instructions: “Focus your attention on *moving the relevant finger* on your left or your right hand as fast and as accurately as possible.” Those in the control group were instructed to “focus your attention on the task at hand.” Before each block of 20 trials, the participants were reminded of the instructions. The participants in the external focus group were reminded to “remember to focus your attention on *pressing the relevant key* on your keyboard (“f” or “j”) as fast and as accurately as possible.” The participants in the internal focus group were reminded to “remember to focus your attention on *moving the relevant finger* (on your left or your right hand) as fast and as accurately as possible,” and those in the control group to “remember to focus your attention on the task at hand.”

After the completion of the practice stage, the participants were asked: “On a scale of 1 (not at all) to 10 (all the time), how well were you able to follow the instructions on how to focus your attention?” After the participant marked down the answer to this question, the software automatically started a 3-min rest. Following this short rest, all the participants performed a post-test that consisted of 20 trials from each of the tasks in a counterbalanced order. No attentional focus instructions were provided prior to or during the post-test. The purpose of the post-test was to examine whether the attentional focus instructions given during practice had any effect on performance when they were no longer provided. After the post-test was completed, the participants were asked again about their ability to follow the attentional instructions.

### Data Analyses

The Kolmogorov-Smirnov test showed that the RTs in the Simon task did not deviate from normality, but that the RTs in the choice-RT task did deviate from normality. However, when examining the skewness and kurtosis values of the mean RTs for both tasks in practice and in the post-test, only one deviation from normality was noted (kurtosis of choice-RT during practice = 2.3). Therefore, we decided to analyze these data with parametric statistics, by performing a two-way ANOVA (Group X Task) with repeated measures on the Task factor to assess differences in RTs in both practice and the post-test. Bonferroni *post hoc* analyses and 95% CIs were used for *post hoc* testing, when necessary. In cases where the *p* value was over 0.05 but under 0.10, and at the same time the effect size was moderate or above (Cohen’s *d* ≥ 0.5 or *ƞ*^2^*_p_* ≥ 0.06), we considered this finding as practically significant and discussed it as such.

Data for correct responses were not normally distributed, and since there is no non-parametric test equivalent for a two-way ANOVA, we performed the Kruskal-Wallis test to assess differences between groups in the number of correct key presses in practice and in the post-test for each task separately. Finally, we intended to repeat the abovementioned analyses for the 25% of participants who reported the highest adherence to instructions and for the 25% of participants who reported the lowest adherence to instructions. This was not possible, however, because more than 25% answered the value of “10” when asked to report the extent to which they were able to follow the attentional instructions, and less than 25% reported the value of “7” or under. Therefore, we did not run this analysis, although it was included in the pre-registration. Finally, we conducted an exploratory analysis (that was not part of the pre-registration) of the performance of both tasks during practice by conducting two-way ANOVAs (Group X Block). All statistical analyses were performed on SPSS version 25, and alpha was set at 0.05.

## Results

**Figure 2:**
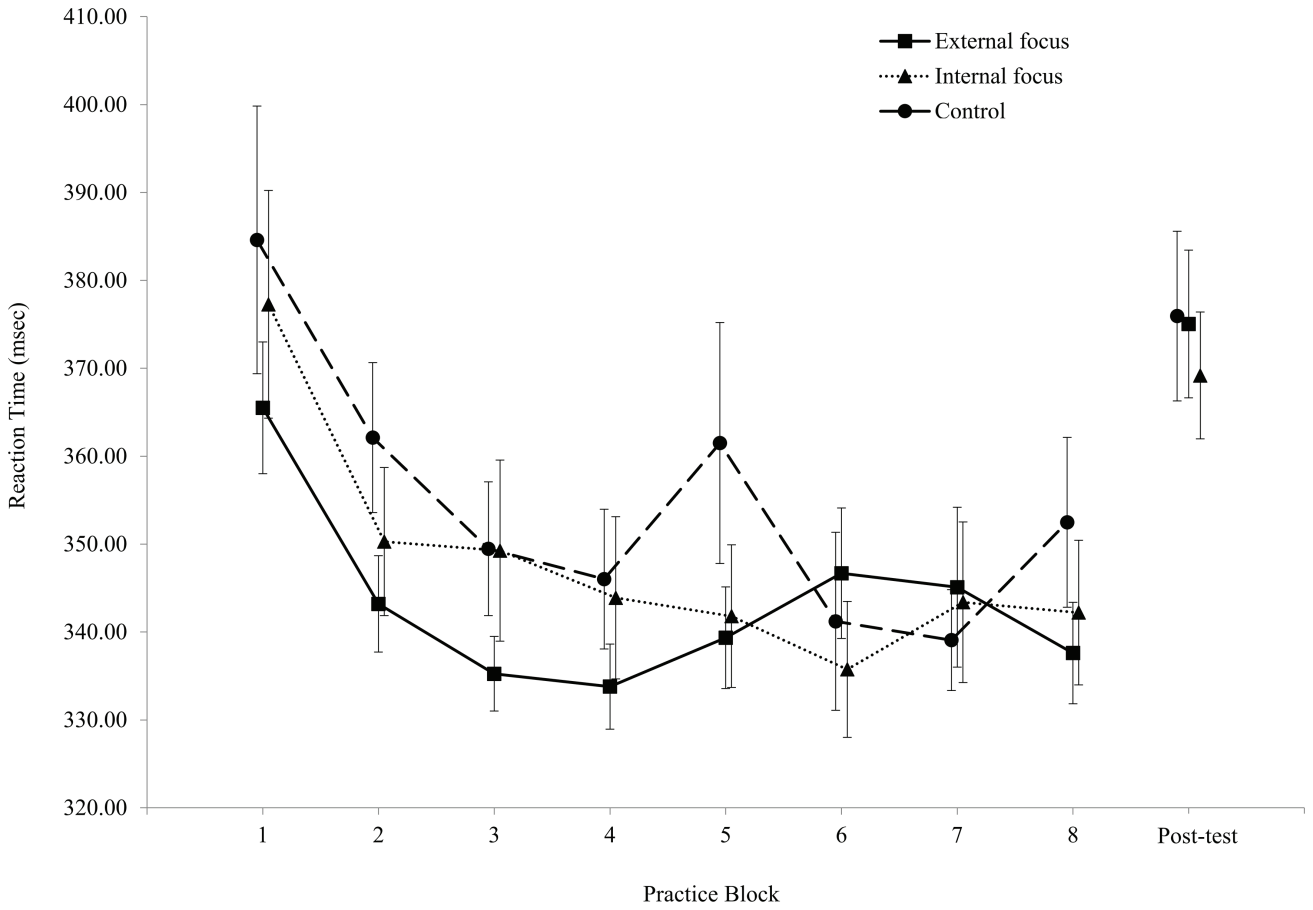
Reaction time in the choice-RT task for all three experimental groups in practice and in the post-test (error bars = SE).

**Figure 3:**
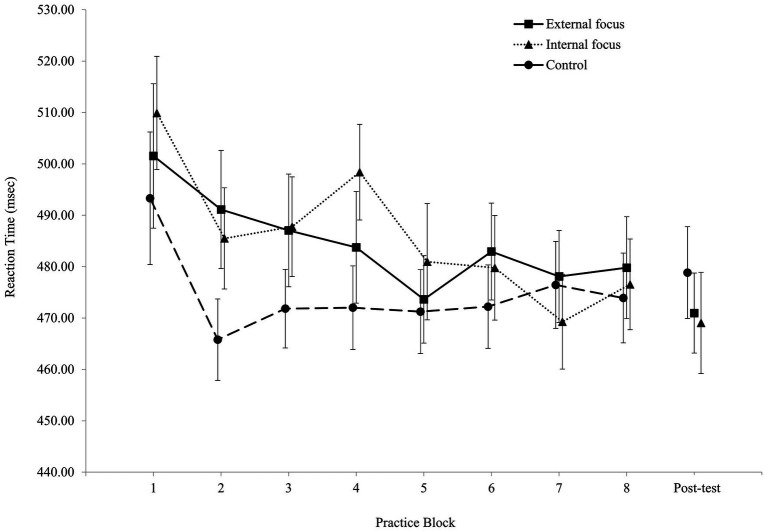
Reaction time in the Simon task for all three experimental groups in practice and in the post-test (errors bars = SE).

Figures 2, 3 present the RTs during practice and during the post-test for the choice-RT task and for the Simon task, respectively.

### Practice

#### Reaction Times

The two-way ANOVA (Group X Task) revealed a main effect for Task, *F*(1, 134) = 1207.28, *p* < 0.01, *ƞ*^2^*_p_* = 0.90. The mean RT in the choice-RT task (348.74 ± 45.62 ms) was shorter than the mean RT in the Simon task (481.68 ± 53.71 ms). There was no main effect for Group, *F*(2, 134) = 0.06, *p* = 0.94, *ƞ*^2^*_p_* = 0.00. The mean RTs for the external focus group, the internal focus group, and the control group were 414.02 ± 38.12, 417.00 ± 49.73, and 414.58 ± 45.04 ms, respectively. Finally, there was no interaction, *F*(2, 134) = 3.03, *p* = 0.052, *ƞ*^2^*_p_* = 0.04. Mean RTs for the choice RT task and the Simon task, respectively, were: for the external focus group – 343.31 ± 31.35 and 484.74 ± 53.84 ms; for the internal focus group – 347.98 ± 50.43 and 486.02 ± 59.25 ms; for the control group – 354.56 ± 51.80 and 474.59 ± 48.01 ms.

#### Correct Responses

An independent-samples Kruskal-Wallis test did not reveal any differences between groups in the choice-RT task (*H* = 3.56, *p* = 0.17) or in the Simon task (*H* = 0.55, *p* = 0.76). The median for correct responses in the choice-RT task was 19.75 (out of 20), with a range of 18.25–20, and the average success was 98%. The median for correct responses in the Simon task was 18.63, with a range of 12.13–20, and the average success was 92%.

### Post-test

#### Reaction Times

The two-way ANOVA (Group X Task) revealed a main effect for Task, *F*(1, 134) = 336.51, *p* < 0.01, *ƞ*^2^*_p_* = 0.72. The mean RT in the choice-RT task (373.39 ± 57.13 ms) was shorter than the mean RT in the Simon task (473.02 ± 60.10 ms). There was no main effect for Group, *F*(2, 134) = 0.32, *p* = 0.73, *ƞ*^2^*_p_* = 0.01. The mean RTs for the external focus group, the internal focus group, and the control group were 423.00 ± 45.02, 419.12 ± 52.52, and 427.39 ± 50.93 ms, respectively. Finally, there was no interaction, *F*(2, 134) = 0.14, *p* = 0.87, *ƞ*^2^*_p_* = 0.00. Mean RTs for the choice RT task and the Simon task, respectively, were: for the external focus group – 375.05 ± 55.70 and 470.96 ± 51.72 ms; for the internal focus group – 369.19 ± 48.94 and 469.05 ± 66.80 ms; for the control group – 375.94 ± 66.19 and 478.84 ± 61.30 ms.

#### Correct Responses

An independent-samples Kruskal-Wallis test did not reveal any differences between groups in the choice-RT task (*H* = 1.81, *p* = 0.41) or in the Simon task (*H* = 1.07, *p* = 0.59). The median for correct responses in the choice-RT task was 20 (out of 20), with a range of 17–20, and the average success was 99%. The median for correct responses in the Simon task was 18, with a range of 10–20, and the average success was 89%.

### Exploratory Analysis

This section presents separate two-way ANOVAs (Group X Block) with repeated measures on the Block factor for the choice-RT task and the Simon task. This analysis was not pre-registered, however, it provides additional insight on performance during practice.

#### Choice-RT Task

The two-way ANOVA (Group X Block) revealed a main effect for Block, *F*(4.9, 660) = 10.25, *p* < 0.01, *ƞ*^2^*_p_* = 0.07. RT in Block 1 (376.01 ± 84.16 ms) was slower than the RTs in the rest of the blocks (between 341.38 ± 51.52 and 352.07 ± 52.10 ms). There was no group effect, *F*(2, 134) = 0.70, *p* = 0.50, *ƞ*^2^*_p_* = 0.01, nor an interaction, *F*(14, 938) = 1.03, *p* = 0.42, *ƞ*^2^*_p_* = 0.02.

#### Simon Task

The two-way ANOVA (Group X Block) revealed a main effect for Block, *F*(4.9, 648.3) = 5.31, *p* < 0.01, *ƞ*^2^*_p_* = 0.04. RT in Block 1 (500.95 ± 85.41 ms) was slower than the RTs in the rest of the blocks (between 474.68 ± 59.89 and 480.41 ± 66.77 ms), except for Block 3 (482.09 ± 64.17 ms). There was no group effect, *F*(2, 133) = 0.48, *p* = 0.62, *ƞ*^2^*_p_* = 0.01, nor an interaction, *F*(14, 931) = 0.72, *p* = 0.76, *ƞ*^2^*_p_* = 0.01.

## Discussion

The purpose of the current study was to examine the effects of external and internal attentional focus on the performance of two tasks: a choice-RT task and a Simon task. We hypothesized that instructing participants to use an external focus of attention would lead to improved performance compared with internal focus instructions or no instructions. However, the findings obtained in our study did not support our hypothesis.

It is possible that the simple RT-based tasks in the current study were not sensitive to the attentional focus manipulation, since in such simple tasks there are not many actions that internal focus can disrupt. It is also possible that the superiority of external focus over internal focus will be more pronounced as tasks become more difficult and require more motor control processes or have more degrees of freedom. In this respect, one study that examined the effects of attentional focus as a function of task difficulty found that the benefits of adopting an external focus of attention were found only in a more difficult balancing task, and were nullified during the performance of a simpler balancing task (Wulf et al., 2007). However, at least in one study (Porter and Anton, 2011) external focus of attention, compared with internal focus, led to better performance in a rotary pursuit task. While this task is different from RT-based tasks, it is still a simple motor task. In summary, despite some previous work on attentional focus and task difficulty (e.g., Wulf et al., 2007; Raisbeck et al., 2020), the role of task difficulty as a moderator of the effects of attentional focus on performance is as yet unclear. Therefore, in future research task difficulty needs to be systematically examined as a moderator of the effects of attentional focus on performance. For example, we can modify the choice-RT or the Simon task used in our study by asking participants to place their fingers on the space bar before each trial. This way, we can add aiming to the task and increase its difficulty.

Another possible explanation is that the participants did not follow the given instructions. Although, we asked the participants to what extent they followed the instructions, we cannot say whether their answers represent their actual attentional focus when performing the tasks. This is a problem in both laboratory and online studies. One way to address part of this problem is to use an instructional manipulation check (Oppenheimer et al., 2009). An instructional manipulation check allows researchers to assess whether participants are reading the assigned instructions. Such checks can be particularly useful in online studies, in which researchers do not interact with the participants and have no way of knowing to what extent the instructions are being followed. For example, at some point during an online study, participants see a screen of instructions with a large “next” button. The researchers are worried that participants will click the “next” button without reading the instructions. Therefore, the instructions are written in a way that, for example, the participants are told to click on the heading of the instructions to proceed instead of the “next” button. In this way, only the participants who have read the instructions are able to proceed. Such manipulation checks ensure that participants read the instructions, but they do not ensure that they use the assigned attentional focus. To examine the latter, it is suggested to implement a dual-task paradigm when applicable and/or an electroencephalogram (EEG; see Wang et al., 2021 for an example of the relationships between EEG and attentional focus).

### Limitations of the Current Study

The fact that we did not include the abovementioned checks is one limitation of the current study. Future studies should include one or more instructional manipulation checks and base their data analyses on the results of these checks. Another limitation of the current study is that the task may have been too easy, and thus there was not much room for improvement. Indeed, in the exploratory analyses of RTs over the eight practice blocks in each task, there was an improvement after the first block that mostly plateaued during the rest of the practice blocks. A third limitation is that we should have asked the participants in a more direct way about whether or not they followed the attentional instructions. Specifically, the question “On a scale of 1 (not at all) to 10 (all the time), how well were you able to follow the instructions on how to focus your attention?” may have been too general, and participants may have answered that they followed the instructions to press the keys as fast and as accurately as possible rather than following the instructions to concentrate on their fingers (internal focus) or on the keyboard keys (external focus). Finally, in such an online study there is no control over the environment or equipment of the participants as they perform the tasks. However, large sample sizes that are more easily attained in online studies can make up for such a lack of control (Woods et al., 2015).

### Strengths of the Current Study

One strength of the current study is the large sample size (*N* = 137). Many studies in the field of motor learning include relatively small sample sizes. For example, meta-analysis of Logan et al. (2012) on motor skill interventions in children reported sample sizes of 12–42 participants per study. Similarly, meta-analysis of Lebeau et al. (2016) on Quiet Eye and performance reported sample sizes of up to 50 participants per study. Depending on the methodology used, these relatively small sample sizes can lead to low statistical power. In contrast, using an online platform can allow researchers to reach more participants with relative ease, thus enabling them to conduct studies with ample statistical power.

Another advantage of conducting studies online is the blinding of both participants and experimenters (i.e., double-blind studies). Laboratory-based studies in motor learning are often single-blinded because the researchers who interact with the participants are aware of their own hypotheses and expectations. This lack of blindness threatens the internal validity of the study (Thomas et al., 2015). In a computerized online study, all the participants interact with and receive instructions from a computer program, with no interaction with the researchers. This ensures double-blinding and strengthens the internal validity of the study.

## Conclusion

In conclusion, the results of the current study show that attentional focus instructions do not affect the performance of a simple choice-RT task or of a Simon-task in a computerized online study. Online studies can help achieve adequate statistical power, and can prevent bias caused by the lack of blinding. We encourage researchers to complement their laboratory-based research with online research.

## Data Availability Statement

The study presented in this study can be found in online repositories. The names of the repository/repositories and accession number(s) can be found at: https://osf.io/gctdf/.

## Ethics Statement

The study involving human participants was reviewed and approved by the Ethics Committee of the Academic College at Wingate. Written informed consent was not provided because this was an online study. Participants filled an electronic informed consent as was approved by the Ethics Committee and as written in the manuscript.

## Author Contributions

GZ: conceptualization, methodology, formal analysis, and writing – original draft. RL: conceptualization and writing – reviewing and editing. Both the authors contributed to the article and approved the submitted version.

### Conflict of Interest

The authors declare that the research was conducted in the absence of any commercial or financial relationships that could be construed as a potential conflict of interest.

## Footnotes

^1^https://aspredicted.org/

^2^https://aspredicted.org/im3v5.pdf

^3^https://osf.io/gctdf/

^4^www.gorilla.sc

^5^www.prolific.co
